# The Prophylaxis and Treatment of Prolapse of the Uterus1Read before the Clinical Society of the New York Post-Graduate Medical School and Hospital, January 17, 1908.

**Published:** 1908-05

**Authors:** James N. West

**Affiliations:** Professor of Diseases of Women, New York Post-Graduate Medical School and Hospital


### The Prophylaxis and Treatment of Prolapse of the Uterus[1]

By JAMES N. WEST, M. D.,

Professor of Diseases of Women, New York Post-Graduate Medical School and Hospital.

[*Post-Graduate*, February, 1908]

THE need of skilled obstetricians and gynecologists has never in the history of the world been so great as that of the present time.

With the increased development of the brain, which comparative ethnology shows to have taken place in the civilised nations, and the consequent increase in size of fetal heads, there has been, unfortunately, no corresponding development of the pelvis to accommodate them. On the contrary, the habits of women in civilised communities are such as to add to the difficulties of parturition caused by large heads, those of small and more or less contracted pelves. To these obstacles to labor is still further added that of the more or less increase in rigidity of the muscles as well as of the articulations of the pelvic bones due to late child bearing. The young savage maiden begins to breed as soon as she has reached puberty, while among civilised nations breeding begins often at a much later period.

Thus Nature tends to hold to a certain type and measure, for the larger the fetal head and the more imperfect the maternal mechanism of reproduction, the greater will be the fetal as well as the maternal mortality in parturition, and also the more frequent will be maternal injuries; and secondarily the establishment of pathological conditions which will prevent future conceptions. The future promises no cessation of these difficulties but rather their increase.

Aside from the motive of humanity which calls for relief from the dangers and suffering incident to parturition, is that of the necessity of the race to preserve the type of the *Genus Homo* with the big brain. This task is the one assumed by obstetricians and gynecologists, and as civilisation advances their work extends and becomes of greater and greater importance.

Among the disabling and painful conditions which may be traced chiefly to the injuries of parturition, prolapse of the uterus is one of the most striking, complicated and difficult to cure; and as in other branches of medicine, prevention offers by far the broader and more useful field of endeavor. It is as a rule so very much easier to arrest a prolapse in its early stages and to prevent its occurrence than to cure it when once established, that it is chiefly the hope of doing some good by calling attention

1. Read before the Clinical Society of the New York Post-Graduate Medical School and Hospital, January 17, 1908.

to this fact which has tempted me to present this subject to you. In order that the statements which follow shall be clearer, I shall present in brief my conception of the forces which maintain the uterus in its normal position.

There are two sets of direct supports; first, those which suspend the uterus and other pelvic organs within the pelvis, the broad ligaments extending from side to side, the utero-sacral ligaments and subvesical fascia which form a ligamentous chain from before backward, and the round ligaments which aid in maintaining the uterus in its forward position. Secondly: The muscles and fascia which close the pelvic outlet and act as the buttress or reinforcement of the intrapelvic supports; of these the most powerful and important are the levator ani muscle and fascia, which in turn are reinforced by another buttress consisting of the muscles, fascia and connective tissue of the anterior part of the perineum which lies anterior to and below it, chiefly the sphincter vaginæ and the transversus perinei muscles with their accompanying fascia and connective tissue. Finally, the intra-abdominal pressure which, acting on the posterior surface of the uterus, holds it forward so that when the bladder is empty the uterus lies in a position almost parallel with the axis of the outer part of the vagina. It is upon the integrity of these forces that the maintenance of the uterus in its normal position depends. It is not necessary that there should be an actual solution of continuity in any of the structures constituting the ordinary support of the pelvic organs, but a superimposed weight may be too great for their natural strength, or a loss of tone in the structures themselves may be such as to cause them to fail in their function of supporting organs of normal weight.

In the female pelvis there occurs a distribution of plain muscular fibres which imparts a peculiar property to the connective tissue so freely distributed here and plays an important part in the changes which occur during pregnancy and in the act of parturition and in the processes of involution which take place afterward. It is a well established fact that nonstriated muscular fibres are found in the broad ligaments, the uterosacral and the ovarian, while the round ligaments are practically composed of them.

In regard to the distribution of the pelvic connective tissue, Von Rosthorn states that the cervix is its center, from which processes of more or less dense connective tissue radiate in different directions in the midst of the more lax connective tissue. It extends to the sides of the pelvis through the basal portions of the broad ligaments, to the posterior portion through the utero-sacral ligaments and to the anterior through the vesico-vaginal septum. Waldeyer states that the relations of the pelvic visceral connective tissue are very important, especially that portion which extends from the posterior inferior portion of the bladder and which is very strongly developed. It runs along the base of the broad ligaments and unites the paracystin, paraproctin, and especially the parametrium and paracolpin with the lateral walls of the pelvis. This is the most complete and terse statement of the arrangement of the pelvic connective tissue which has ever come under the observation of the author.

The existence of fascia beneath the bladder and above the vagina has been positively denied by some authors and equally positively affirmed by others. I have repeatedly gathered up and stitched there what I believe to be a continuation of the recto-vesical fascia which forms a slinglike process beneath the bladder, being an offshoot from the pelvic fascia at the sides and attached to the cervix behind and the pubes in front. It is this fascia which, together with the utero-sacral ligaments, forms the antero-posterior ligamentous chain which is such an important element in sustaining the uterus. The great importance of an appreciation of these supporting structures and of the connective tissue especially, *i.e.,* aside from the muscles, is tersely put by Savage, who says: "Permanent cure of uterine prolapse depends chiefly upon the elastic qualities of subperitoneal pelvic connective tissue. The latter always retains its relation with the displaced organs as well as with the pelvic vessels. Owing to the slow progress of the prolapse, the connective tissue yields to an enormous extent; but the fibro-elastic elements of its structure will very often enable it to return eventually to its normal condition if relieved from the weight of its prolapse."

The conditions which favor overstretching of the pelvic connective tissue and prolapse are:

1. A solution of continuity of greater or less degree of the muscles and fascia of the pelvic floor.

2. Conditions which cause loss of tone or resiliency, as inflammatory conditions, the presence of necrotic material in the uterus obstruction to the local circulation, and constitutional conditions.

3. Those which cause an increase of weight in the pelvic organs beyond that which their supports were intended to bear, as tumors or subinvolution.

4. Alterations in the position of the uterus in regard to the direction of its long axis and consequent alteration of the effect of intraabdominal pressure. All or any of these conditions may exist in combination.

The provision of Nature whereby a large canal leading from within the body may remain closed when not performing its function and closed with such firmness that no descent of the intraabdominal contents occurs, is that of oblique penetration. The inguinal canals offer an example of this, the course of the canal being oblique, the intraabdominal pressure is met at every point by the full thickness of the abdominal wall. In the example of the vagina, the opening of which lies directly under the pubic bone, the axis of the canal is such that the intrapelvic pressure is at right angles to, and its full force is met at every point by, the powerful levator ani muscle and fascia and the muscles of the perineum.

When the levator ani and perineum, one or both, are lacerated, the direction of the intraabdominal force becomes more or less straight instead of oblique and the lower support being removed the whole strain falls on the intra-pelvic ligaments and connective tissue. When these begin to stretch and give way the cervix swings further in toward the pubes and the fundus backward and the first long stride toward procidentia has begun.

The first important step, then, in prophylaxis in such cases is early and thorough repair of the perineum and posterior vaginal wall. This principle is recognised by many but the details of the operation are often carried out so poorly that the patient is worse off than if no operation had been done, for she now is placed in that position of false security brought about by the delusion that her injury has been repaired. From a very extensive experience in teaching plastic surgery on the living subject I am convinced that no common operation is so little understood, and meets with such uniform failure as that of repair of laceration of the perineum. I try to have my students forget the names of operations but to study the nature of each laceration presented and to repair it in accordance with the conditions found and with the appropriate suture materials, and the application of the proper mechanical principles in using them and almost equally important, the proper treatment of the cases after they have been operated on.

"Conditions which cause loss of tone or resiliency, as inflammatory conditions the presence of necrotic material in the uterus and obstructions to the local circulation and constitutional conditions."

Here alone is a vast useful field for prophylaxis.

The inflammatory conditions referred to are particularly those of mild degree such as may be set up during the healing of a lacerated cervix or the presence of endometritis fungosa, and last but most important, the presence in the uterus of the products of conception imperfectly discharged.

I believe that every case of abortion should be curetted, have the uterus thoroughly washed out and painted with pure carbolic as soon as it is positively ascertained that abortion is inevitable and the cervix has become dilated. This should be done as a regular operation under an anesthetic and with the most rigid asepsis carried out. Proper attention to the patient afterward is here of very great importance. Thorough emptying of the uterus in labors at term need hardly be mentioned here, for if this is neglected and portions of placenta are left behind the patient will hardly live to have a prolapse.

Under the head of obstruction to the local circulation, aside from laceration of the posterior vaginal wall and perineum, laceration of the cervix is a most frequent cause also, inducing in a great number of cases endometritis fungosa. Such lacerations as are of sufficient extent to cause symptoms should be repaired within the first six months after their occurrence and in most instances should be accompanied by a light curettage. Such operations are by no means easy of performance and many of them require a high degree of technical skill.

Aside from the local causes mentioned which may cause loss of tone and prolapse, are constitutional causes and occupation. Certain cases where no injury of any kind has occurred show prolapse. This in many instances is undoubtedly due to the general lack of tone throughout the body and to poor nourishment and poor hygiene.

The statistics of the German clinics tell us a pathetic tale of unsuitability of women to do the heaviest work. Nearly every German clinician states that this condition is found chiefly among the laboring classes and those especially who have great burdens to bear or to make great physical exertion.

Döderlein and Krönig state that in the material of the Türbinger clinic, prolapse forms 12½% of the cases and gives the most frequent indication for operative interference.

It occurs most frequently between the ages of 36 and 56 years and only 2% of the prolapse cases in this clinic had not borne children. Then with the first symptom of prolapse in those of a general atonic condition, in addition to a local support such as a pessary, every effort should be made to promote the general welfare of the body, fresh air, nutritious, simple diet, exercise, massage and proper medicinal tonics, of which strychnine should form the most important element. Where the prolapse appears to be due to occupation this should be changed if possible and a pessary applied. The use of the pessary finds its best field in early prolapse cases, but those who wear pessaries should be under the constant observation of a physician who understands their use.

"Those conditions which cause an increase of weight in pelvic organs beyond that which their supports were intended to bear as tumors or sub-involution."

The prophylaxis in the case of tumors is to remove them and that for the cause of sub-involution has already been dealt with under other headings. In addition to the appropriate surgical measures of repair of the cervix and curettage may be mentioned constitutional and local treatment; under the former may be included measures of hygiene as proper exercise and fresh air, massage and medication; under the latter medicated tampons and hydrotherapy almost any of these subjects would be alone a theme for a paper.

"Alterations in the position of the uterus in regard to the direction of its long axis and consequent alteration of the effect of intraabdominal pressure and resistance."

This heading included most prominently retroversion in its varying degrees, and presents probably the most fruitful field for prophylaxis.

Retroversion is almost always a necessary stage in the course of the formation of prolapse which leads to procidentia. A very large proportion of the retroversions following labor or abortion are easily preventable.

T. A. Emmet says that procidentia may be due to an enlarged uterus remaining settled from some cause on the floor of the pelvis after labor. The author has found retroversion in his clinic most frequently to be due to the uterus having settled back into the hollow of the sacrum after labor. If this displacement is corrected early and the uterus held forward by an artificial support, such supports need be, as a rule, only temporary, for by their union with other measures to promote involution the normal tone of the connective tissue and ligaments may soon be restored and the uterus naturally remains in its proper position.

Directly in proportion to the duration of time from the occurrence of the displacement do the chances for restoration by such palliative measures diminish. It is this form of retroversion added to a moderate tear of the levator ani and fascia which most frequently leads to procidentia. A woman may endure this condition comparatively well until the climacteric, when the atrophy of tissues which had until then been strong enough to hold the uterus up occurs and in a comparatively short time procidentia will occur. Should we then fail by palliative measures to cure a retroversion, a suitable operation should be performed.

Every woman after confinement at term or abortion should be examined several times at intervals of about five days to ascertain whether the uterus is coming to its proper position or not.

I prefer to make my examinations, ten, fifteen, twenty and twenty-five days after labor. If all has gone well until then displacement is not apt to occur. This, as I have said before, is, I believe, one of the most fruitful fields of prophylaxis against prolapse and procidentia. The scope of one paper will only admit of a very condensed presentation of this subject and I therefore proceed to a brief consideration of the treatment of procidentia.

We must recognise at once that since this is often a disease of the aged and infirm, and becomes more distressing as age advances, there must be many cases which on account of general infirmity and complicating diseases are far beyond the reach of operative interference. Such cases must of necessity be treated in a palliative way. Here we must resort to decubitus, pessaries, balls, and etc. To me the most generally satisfactory instrument has been the hard rubber ring with large calibre and comparatively small opening. The hard rubber discs with small openings in the center have proven too irritating. The large rubber ring with small calibre is dangerous. The whole uterus and vagina may prolapse through such an instrument and undergo gangrene. The author only averted this with a patient by sawing through such a ring at its most dependent point with a wire saw. protecting the parts beneath with a thin spatula. However, with all the care and ingenuity it is most difficult to make such patients comfortable.

The cases which are operable have called forth the greatest ingenuity and the most strenuous endeavors for their relief.

An absolutely satisfactory procedure has not yet been devised and this fact is emphasised in the more recent efforts of German surgeons for whom the problem seems to be an even more serious one than for us here in America.

Döderlein and Krönig give an interesting history of some of the more radical measures of which I shall herewith present an epitome.

"Hysterectomy for prolapse was performed by Langenbeck in 1813, Gebhardt 1836 and 1837, Jürgensen 1838, Edwards 1864. Choppin for the indication of prolapse only in 1867. Kaltenbach encouraged by Czerney's vaginal extirpation for carcinoma placed the operation for procidentia on a firm basis; since after extirpation of the uterus the loosened vagina fell forward, Kaltenbach performed anterior and posterior colporrhaphy at another sitting as a conclusion.

"Fritch later removed in the worst cases also the greater part of the vagina with the uterus. Martin recommended extirpation of the uterus and the whole vagina for the worst cases. At a gynecological meeting in Frankfurt the following operators accepted total extirpation of the uterus and vagina as the proper procedure under conditions which follow: Bouilly, Richelot, Terrillon, Gouillioud, Pozzi, Quesnu, Ségond, and Lejos; provided (1) that the patient should be in or beyond the climacteric.

"2. The totally inverted vagina should contain the totally prolapsed uterus.

3 The vagina has upon it in addition to great hypertrophy multiple ulcers.

"4. Excluding as contra-indications those in very old age or having complications of severe illness."

The author does not feel that the above view should be accepted as he has never seen a case of procidentia where the proper treatment which will be briefly described later would fail to heal the ulcers and reduce the hypertrophy after which other less radical and much more satisfactory measures could be used. Also some of the most miserable human beings the author has ever seen have been women upon whom the removal of the vagina, uterus and ovaries has been done for prolapse.

The author believes that a properly performed Lefort operation with the appropriate plastic work accompanying it would meet the indications in any case in which the above mentioned operation might be considered by its advocates to be suitable and without the evil consequences.

*Operation of Freund.*—This was devised in order to use the fundus of the uterus as a plug in the vagina to help to prevent its own descent. In this one, incision is made in the posterior vaginal fornix and the uterus is revolved backward upon its axis and the fundus brought down into the vagina. The uterus is sewed in this position to both anterior and posterior vaginal walls and an opening made in the fundus for drainage. This is not extensively endorsed in Germany. The former operation of Freund consisting of successive rings of silver wire buried beneath the mucous membrane of the vagina has proved to be a failure.

The author can not conceive that either of these operations should ever be performer.

The Schauta-Wertheim is a modification of the Freund. They dissect the bladder well away from the anterior vaginal wall, make an incision through the peritoneum at its utero-vesical fold and bring the uterus forward, stitching it under the bladder and partially covering it with the flaps of anterior vaginal wall, completing the operation by a posterior colpoperineorrhaphy. This allows the cervix to drain into the vagina and leaves this canal still patulous.

The latter operation is one of the recent developments in Germany of the treatment of procidentia.

I trust that I may be pardoned for the criticism which seems to me to be almost presumptuous, but I can but feel that all those destructive operations and deforming operations which involve the placing of organs in abnormal positions are based upon wrong principles, and that the greater the extent to which they are followed out the greater the sum total of human sufferings. We well know the distressing symptoms produced by displacements of the uterus of the ordinary type, and experience has taught me, through the study of some of those unfortunate subjects of deforming operations that the suffering produced by the operation is actually greater than that caused by the original disease.

Complications may make hysterectomy necessary in some cases, but as an operation of preference I could never consider it. In America the weight of the best opinion also favors this view. In the course which I pursue in the bad cases a long preparation by rest in bed with the foot elevated, and copious astringent hot douches forms an essential feature. To this should be added scientific massage, a good and nutritious diet, and a tonic of which strychnine shall be the chief basis.

Under this regimen ulcers of the vagina or cervix will heal, involution will take place in all the hypertrophied structures, and the pelvic connective tissue will in a large measure regain its tone and the organs their natural relation.

When the maximum amount of benefit has been attained the patient is ready for operation. A complete detailed description would carry this paper to too great a length. I would simply say then, that in most cases the levator ani and perineum are carefully repaired, the anterior wall has a radical opertion made upon it, often the cervix is amputated and ventral fixation of the uterus made. Some variations are sometimes made but it is my good fortune to have cured, absolutely, a fair number of very bad cases of complete procidentia without using any of the destructive deforming operations of which I have had some opportunity to see the bad results.

The details of the preliminary preparation and of the plastic operations and the fixation of the uterus and the question as to whether the patient should be rendered permanently sterile are all of the utmost importance and would form in themselves a subject large enough for a separate paper. I will therefore close with a strong appeal for a more thorough prophylaxis and a rational operative procedure, which shall have for its chief aim a restoration to natural conditions.

71 West Forty-ninth Street, New York.

DISCUSSION.

Professor Ward.—If I understand Dr. Waldo correctly, his operation is to produce a marked anteversion of the uterus. His method of opening the anterior vaginal wall is very familiar to me. It has been advocated by Dr. Goffe of this city very extensively. In this way diseased organs are attacked through the anterior rather than the posterior fornix. Making a T-shaped incision and anteverting the uterus through the wound is not difficult. As an illustration of this I will mention a case where I was curetting a uterus some years ago. The uterus was very soft and the walls very thin, following miscarriage, and the curet went through the fundus without my realising it. It was a private case and I knew that the wound should be repaired at once as the curet was a large one. The expedient of making a T-shaped incision, as suggested by Dr. Waldo, was very easily followed out in this case, the uterus anteverted into the vagina and the rent repaired. Cystocele, which is hernia of the bladder, can be cured by this same procedure, advocated by Dr. Waldo for prolapse of the uterus. I do not rely upon the Sims, or Stoltz operations, or other of that type for the cure of cystocele operation, but believe we must first free the bladder from vagina and uterus and then attach it higher up. Noble, at the American Medical Association, last June, gave credit to Dr. Hadra of Texas, who was the first to recommend freeing the bladder and fastening it higher up.

Subinvolution is one of the most frequent causes of procidentia. Subinvolution applies to the vagina, which has been stretched in allowing the child to come through, and to the ligaments, which have been stretched in carrying the child to term, as well as to the uterus. With slack ligaments, a large, heavy uterus, and a lax vagina we have all the conditions which would tend to cause the uterus to fall out. The blame, in many cases, so far as prophylaxis is concerned, is traceable to something going amiss during the child-bearing function. It is the man who puts on forceps without remembering that in so doing he must closely imitate Nature, who is responsible—that is, he must complete the first stage of labor before beginning the second. He puts one blade of the forceps through a partially dilated cervix, then the other, then locks them, and tries to bring both of them at once out of the same sized opening. The result is a severely lacerated cervix which leads to subinvolution and its sequelæ. If he lacerates the pelvic floor as well he probably looks at the perineal body and does not see it torn, but fails to look *inside* the vagina, to see if there is a tear in the sulcus.

I am glad to hear Dr. West say he believes in the radical operative treatment for abortion. I read a paper before this Society some years ago in which I recommended treating abortion by the operative rather than expectant plan. I believe abortion is one of the most frequent causes of subinvolution. If a patient gets up, as she usually does, with a heavy uterus and subinvoluted ligaments, she may have prolapse from subinvolution following abortion just as after full term. I believe in making an operation of every inevitable case of abortion, putting the patient under an anesthetic, curetting and washing out the debris.

I agree with Dr. West regarding the preparatory treatment of these cases of prolapse. With rest, nutrition and improvement of the general health, as suggested by him, it is surprising to see the difference in the appearance of the parts after a few weeks. Where we have supposed that it would be impossible to do anything but a radical operation it is often possible to cure the patient by milder means. If a woman is near the menopause we have a choice of methods, none of which are perfect, I have tried nearly all. One which I have done a number of times is to amputate the uterus above the cervix, stitching it to the abdominal wall. We cannot be certain as to the result. Many of these cases are very trying, and the court of last resort, where absolute failure by other methods has occurred, is the operation described by Edebohls, his panhysterocolpectomy. It is only in selected cases, however, where such an operation is advisable

PROFESSOR GRABER.—One of the fundamental questions in connection with procidentia is the causation, why some women have prolapse of the uterus and others do not. In order to understand the causation we must consider the formation of the pelvis. the peculiar inherent condition of the woman's tissues. Some women will not have prolapsus uteri no matter what is done to them. In some women the tissues are such that the perineum may be torn into the rectum and there will be no prolapsus, there being a sort of retraction toward the sacrum which does not permit of the uterus coming down. Again, a woman who is prone to relaxation of her tissues will have a general tendency to prolapse, and the parts may begin to come down long before she gives birth to children—often there is a rolling out of the anterior and posterior vaginal wall in women who have never borne children.

We do not recognise sufficiently the effect or the symptoms that an ordinary descensus will cause. The ordinary first degree requires a great deal more attention than is usually given it. The axis of the vagina, that is, the pelvic outline in cases of ruptured perineum is more or less in line with the abdominal axis, and whatever operation is performed will have to change the vaginal outlet so that there is not that direct pressure downward. In order to accomplish this, every operation will have for its purpose putting the uterus in anteversion. With the uterus anteverted there will be no prolapsus. On the other hand, retroversion is always an accompaniment of procidentia.

With the proper pessaries a great deal can be accomplished for these women, to such an extent that they need not be operated upon in many cases. The large, narrow ring should not be used. The uterus dips into it and gradually goes down and through it until we find it coming out. If, on the other hand, we use a ring which is thick it will hold the uterus in position if there is any perineum at all. Now and then we can tampon with some astringent powder, the tampon to be left in for a week. If relief can be given by a simple procedure of that kind I think it is well enough to employ it. The pessary must be removed every six weeks or three months at most to be cleansed.

As to operative treatment it is well if we can give tolerable assurance that we can give permanent relief. We cannot exactly do that now, but at the same time every operation devised for this condition of affairs will give a certain number of cures. It is by experience, by long, hard work, that we learn what operation in a particular case will accomplish a cure. Suspensions are not successful. Alexander's operation, thought at one time to be a method of cure, after all, is only a factor in drawing the uterus forward and is practical only in partial descension. Some have worked on the utero-sacral ligaments. If we could make a proper utero-sacral ligament we could prevent the uterus coming down, but inasmuch as we have no way of making a ligament strong enough to hold the uterus in position, this method is not a success. The one operation which seems to be most satisfactory is the one described by Dr. Waldo. If we can devise an operation for drawing the uterus in anteversion, close up the outlet and bring the uterus as much forward as possible, we will go a long way toward curing this condition. No operation which will not hold the uterus in an anteverted position will be successful.

Dr. Bandler.—I have had quite an extensive experience in operating by the method described by Dr. Waldo, and I think it offers the best results. Not only does it bring the uterus forward but it puts the bladder upon the first surface of the uterus and so prevents cystocele; at the same time we are able to do an extremely high amputation of the cervix, which leaves a very small uterus. Furthermore, we can excise a large area of the posterior fornix and finally a posterior colporrhaphy, particularly a colpoperineorrhaphy. The bladder disappears from the field of operation, the fundus stays forward, and in a large percentage of cases we have effected a cure. I have had only one failure. An important element in this operation is the small amount of shock. I have just operated by this method upon a woman 65 years old who was returned to bed with a pulse never beyond 72 and who was in splendid condition the next day.

I agree with Dr. West in the essential features of his paper, especially in the matter of prophylaxis. We must watch these cases after labor and should never consider a woman released from observation until the uterus and its ligaments as regards size and position are entirely normal. This can best be accomplished by rest, douches, tampons, pessary, and particularly by correcting subinvolution and retroversion. The instant you have sagging and retroversion you have the elemental stage of procidentia. It is this sagging, or descent, which may well be called hysteroptosis, which brings the cervix downwards and forwards and so causes the fundus to fall backward into retroversion or retroflexion.

PROFESSOR WALDO.—I am glad Professor West brought out the importance of treating these cases in their incipiency and of correcting those conditions which lead up to procidentia. I am also glad to hear him say he *treats* abortions.

In a general way, I should say that pessaries are important, but they are of only temporary benefit.

In the operation of Dr. Goffe he goes in and cuts the peritoneum transversely, which is a very good method where the round ligaments are to be shortened, but he never closes the peritoneum separately. With decided fixation of the uterus subsequent pregnancy will give rise to very serious complications.

I want to emphasize the fact that the first step of procidentia is retroversion, and two other steps that follow very closely on that are rectocele and cystocele. Without those three conditions you will not have procidentia. I have had similar experiences to those of Dr. Ward in cases of complete procidentia. I have performed ventral fixation and suspension, I have tried the Emmet and Stoltz operations for cystocele and the old elliptical incision, and I must say that I have not been very successful. I am not speaking of those cases where everything comes out before the patient is married. No one operation will cure everything. The operation of which I have spoken is the outcome of the work of a number of operators, but the particular method which I have described, and which I have followed rather closely in attaching the uterus anteriorly is that of Professor Dührssen.

PROFESSOR WEST.—Some of the gentlemen have had a tendency to attack the uterus and remove it. and for that reason I have attempted to emphasise prophylaxis. Those who have spoken tonight are in accord on that subject. and also with reference to the method. We have in my service in this hospital a great many cases of procidentia, all operated upon in more or less differing ways according to the case. I have here the uterus removed from a patient who had double pyosalpinx. There was no reason for saving the uterus, it being in a state of complete procidentia. The cervix shows actual hyperplasia; it is at least half an inch longer than the body. We sometimes have cases where the cervix is outside and yet the uterus swings up in almost the normal position.

